# Supplementary material for: LMCSleepNet: A Lightweight Multi-Channel Sleep Staging Model Based on Wavelet Transform and Muli-Scale Convolutions
Source: Sensors (Basel). 2025 Oct 2;25(19):6065. doi: 10.3390/s25196065 (PMC12527093; doi:10.3390/s25196065)
Supplement: Supplementary file 1 [file sensors-25-06065-s001.zip › sensors-3805197-supplementary.pdf]

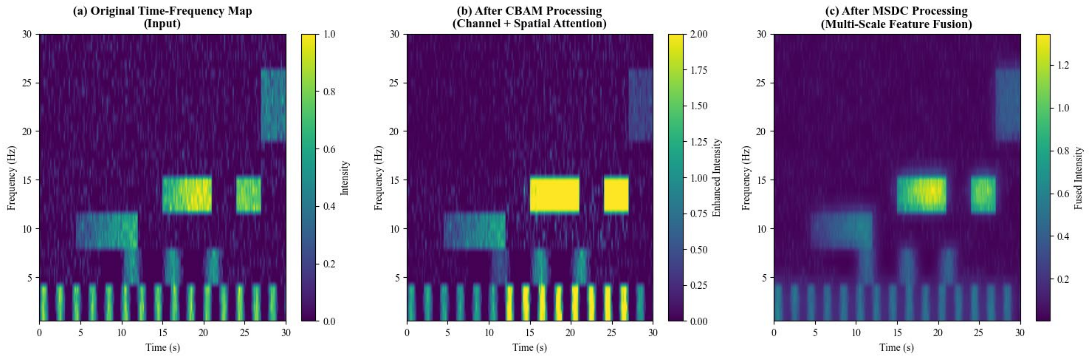

Figure S1 Visualization of Data Processed by CBAM/MSDC

Table S1. Summary comparison between SleepEDF-20 and SleepEDF-78 datasets

**Dataset**	**Subjects (n)**	**Age range (years)**	**Health condition**	**Channels used in this study**	**Sampling rate**	**Total epochs (30 s)**	**Class balance characteristics**
SleepEDF-20	20	25–34	Healthy (SC subset, no medication)	EEG-Fpz-Cz, EEG-Pz-Oz, EOG (horizontal)	100 Hz	43,141	Relatively balanced, but N1 has fewer samples
SleepEDF-78	78	25–101	Mixed (healthy and elderly, no medication)	EEG-Fpz-Cz, EEG-Pz-Oz, EOG (ROC-LOC)	100 Hz	196,350	Strong imbalance: N2 and W dominate, N1 and N3 under-represented

Table S2. Ablation study results of LMCSleepNet on the SleepEDF-78 dataset

ResNet18	CBAM	DSC	MSDC	Acc	κ	MF1	Params/M	W (F1)	N1 (F1)	N2 (F1)	N3 (F1)	REM (F1)
√				83.2	0.75	76.8	11.69	91.1	43.8	84.9	79.2	79.1
√	√			84.0	0.76	77.7	11.71	93.5	46.9	85.6	80.1	82.4
√	√	√		83.4	0.75	76.9	1.48	91.7	42.6	85.0	79.7	79.6
√	√	√	√	84.1	0.77	77.7	1.49	94.2	48.5			

Table S3. Comparison of Single-Segment Training Time of LMCSleepNet on SleepEDF-20 and SleepEDF-78 Datasets

Model	Params /M	SleepEDF-20 Single-Sample Training Time (s)	SleepEDF-78 Single-Sample Training Time (s)
DeepSleepNet	21.0	3.48	4.26
MultiChannelSleepNet	13.0	2.15	2.95
SleepEEGNet	2.1	0.35	1.12
TinySleepNet	1.3	0.22	0.94
SalientSleepNet	0.9	**0.15**	0.84
LMCSleepNet (Ours)	1.49	0.25	0.95
